# Research on shipborne aided navigation system based on enhanced traffic environment perception

**DOI:** 10.1371/journal.pone.0206402

**Published:** 2018-10-31

**Authors:** Yaotian Fan, Liwen Huang, Dan Jiang, Xianzhang Xu

**Affiliations:** 1 School of Navigation, Wuhan University of Technology, Wuhan, China; 2 Hubei Key Laboratory of Inland Shipping Technology, Wuhan, China; 3 Department of Navigation, Chongqing Jiaotong University, Chongqing, China; 4 Faculty of Engineering and the Environment, University of Southampton, Southampton, Hampshire, United Kingdom; Universiti Sains Malaysia, MALAYSIA

## Abstract

The traditional shipborne navigation system uses a two-dimensional electronic chart as a platform to integrate and control a variety of electronic navigation equipment information. It cannot intuitively restore the actual traffic environment or fundamentally improve the perception ability of the crew in poor visibility conditions. This paper proposes integrating three-dimensional simulations and real ship driving systems and presents research on “virtual–real” and “dynamic–static” ship navigation technology and equipment. In the proposed method, the hydrologic factors, topographic features, waterways, traffic dynamics, and ship driving information are effectively integrated, with a focus on achieving key advancements such as the integration of simulations and real ship driving as well as multi-source information fusion. A multi-angle, all-around, multi-level visual display for water traffic environments in poor visibility conditions is provided to enhance the ability of crews to perceive their traffic environments and thereby to provide auxiliary navigation for ships in complex weather conditions.

## Introduction

During ship navigation, the crew faces the natural environment, which includes meteorological factors, hydrologic factors, and underwater terrain, as well as the traffic environment, which consists of waterways, aquatic buildings, beacons, other ships, and the dynamics of the ship itself. These environments contain complex, multi-source information that is necessary for safe operation of the ship. Normally, the collection, perception, screening, analysis, and utilization of the information are isolated and scattered. The crew cannot obtain necessary information about the external environment effectively in conditions with poor visibility, and insufficient judgment of the traffic environment directly affects the sailing safety of the ship. Ship driving simulator technology is already very mature, and it can accurately describe “static” objects such as waterways, hydraulic structures, beacons, shore shapes, topography, and ship shapes using “virtual” methods. Meanwhile, the “real” navigation equipment on the ship can provide dynamic information about the “dynamic” objects such as the ship itself and the target ship. Therefore, the current simulator technology and ship driving systems already have the functions and characteristics needed to combine simulations and real ship driving for auxiliary navigation [1–6].

In the field of shipborne auxiliary system, the navigation and traffic environment information are mainly displayed on the 2-dimensional ECDIS. While the drawback of this 2-dimensional display mode is that the information of multiple key traffic environments cannot be indicated precisely, especially the landscape, aquatic structures and fluids field which are vital for ships on restricted waters such as inland rivers and port. Since the lack of display of the above information would make significant influence on safety and efficiency of ships navigation while visibility is poor, it cannot meet the navigating requirements in that condition. For data fusion of shipborne sensors, the current research mainly concentrates on the track correlation and data fusion of AIS and shipborne Radar. Most research works apply only single numerical model for calculation and processing [7–12]. However, these works may not be very practical for solving the problems of systems’ reliability and stability that probably caused by the high complexity and intensity traffic flow on inland river and ports waters, as well as the frequent turning of ships. From the aspect of system integration, Meng H, Zhao G, and Wang L proposed a semi-physical emulation system of ships motion which can carry out emulation research on ship maneuvering system such as ships course, track, stabilization and collision prevention in laboratory. This work significantly contributes to the prototype of ships motion system controller but it can hardly be applied to ships navigation [13]. Thomas Porathe proposed a conception of 3-dimensional system which integrating information of own ships, other ship, water depth and radar. He also suggested a self-centered 3-dimensional nautical chart [14].

It is thus necessary to develop a shipborne three-dimensional navigation system (STDANS) and to integrate the information about the natural environment, traffic environment, ship dynamics, and navigation systematically in poor visibility conditions. This system will conduct a “virtual–real,” “dynamic–static” combination of simulation and real ship driving, intuitively display the multi-source information closely related to ship driving and navigation safety [15–18], and enhance the ability of crews to perceive their traffic environments in poor visibility conditions, to provide auxiliary navigation for ship driving and improve the safety of ship navigation.

## System elements

As a complex weather phenomenon, poor visibility has certain degrees of frequency, variability, suddenness, and risk. To consider these complex weather characteristics and navigation requirements, the STDANS should have the following characteristics,

(1) Simulation

The most significant differences between navigating in normal weather and in poor visibility conditions lie in the amount of information that the crew can obtain from the traffic environment and its ability to perceive the traffic environment. Therefore, the STDANS should provide the crew with a comprehensive, accurate, real-time, and intuitive view of the traffic environment by generating a realistic simulated navigation environment within a certain range, so that the crew can perceive the real environment to some extent through the system terminal. The use of simulations and 3D visualization technology is required to achieve high-precision characterization of topography, waterways, wading buildings, ships, and other objects.

(2) Fusion

When a ship travels in poor visibility conditions, it can only rely on the shipborne radar and the automatic identification system (AIS) to be informed of the target ship dynamics, and each device has its own advantages and limitations. The radar can continuously track a target, but only at short distances and with low azimuth resolution, and the static features of the target, such as shape, type, and identity, cannot be identified effectively. Meanwhile, AIS can obtain the static parameters of the target ship, but the dynamic data requires a certain period to update. Compared to the sea or open water, internal waterways have many curves, and ships must turn frequently and slow down; processing AIS data alone cannot accurately reflect the ship dynamics. While the data from multiple shipborne sensors overlap and complement each other, multi-source data fusion can be performed to a greater extent to determine the location and dynamics of the target ship. Therefore, the STDANS must be able to perform this fusion.

(3) Integration

The main sources through which the crews of ships with poor visibility can obtain traffic environment information are sensors such as shipborne radar, AISs, global positioning systems (GPSs), compasses, depth sounders, etc. However, the deck officers are unable to meet the requirements for safe sailing on restricted waters such as inland rivers and ports in complex weather conditions by relying only on these types of information. The information these devices present is usually scattered and isolated, increasing the pressure for the crew to receive and analyze the information and make judgments and decisions; meanwhile, with poor visibility the crew cannot effectively visually observe topographic features, aquatic buildings, channel information, hydrographic features, navigational aids, etc., so it is necessary to conduct systematic integration to obtain the above information and to display the information synthetically on the same platform using three-dimensional (3D) simulations and real ship driving technology. Therefore, the STDANS must be capable of integration.

(4) Reliability

The STDANS mainly provides navigation services for poor visibility conditions; consequently, its reliability is particularly critical, which is mainly reflected in the following aspects: (a) the static object location, shape, contour, nature, and other characteristics must be accurately described, and dynamic target movement should be accurately reproduced in real time and (b) systems based on “virtual–real” integration should be validated in real ship environments to ensure their reliability. Thus, the STDANS must be reliable.

### Overall system framework

**Fig 1:**
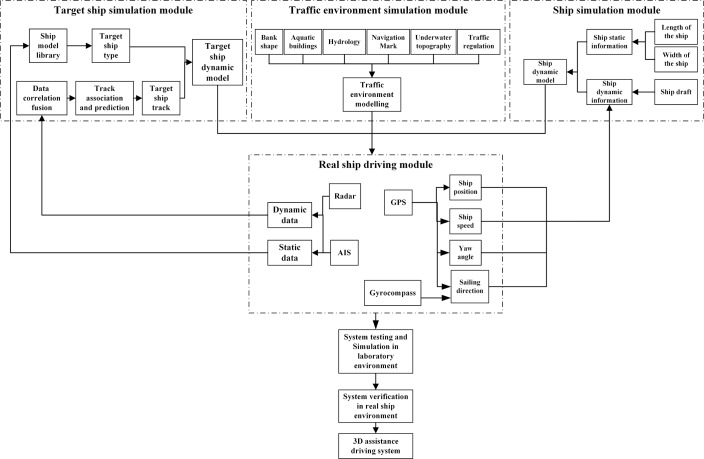
Overall framework of STDANS.

According to the above descriptions, the STDANS should be composed of four modules: traffic environment simulation, target ship simulation, ship simulation, and real ship driving modules. The overall architecture of the system is shown in Fig 1. The functions of the modules are as follows:

(1) Traffic environment simulation module

The traffic environment simulation module generates the land surface topography, bank shape, aquatic buildings, weather information, hydrological information, navigation aid signs, underwater topography, traffic regulation, specific water areas, and so on. The purpose of this module is to provide the deck officers with a reliable image of the environment through precise simulation and reconstruction of the bank line, aquatic buildings, shore scene, navigational aids, and so on, to make up for the limited ability to perceive the relative position of the ship in poor visibility conditions. Furthermore, based on the real-time data such as the water level, flow rate, tidal, etc., the module is to simulate the hydrological values for the water area in which the ship is located. It also presents the flow direction, flow velocity, and water depth in the form of vectors, making up for the limited ability of the driver to perceive hydrological information in strange or variable water areas. In addition, based on the visual description of the underwater topography, traffic regulations, and specific water area, the module displays the channel, anchorage, routing system, reporting line, depth contour, obstruction, reefs and shoals, etc., effectively helping the driver choose the right route.

(2) Target ship simulation module

The function of the target ship simulation module is real-time presentation of the static and dynamic characteristics of nearby ships. By selecting the matching ship model in the model library using static AIS data, the fusion of multi-source dynamic information, such as AIS, GPS, radar, and traffic environment information, is conducted. Target ship track correlation and prediction is conducted, and non-discontinuous, real-time motion simulation of the target ship is achieved, which will increase the accuracy of the target ship real-time data and enhance the ability of the driver to perceive and analyze the static characteristics and motion of dynamic targets in poor visibility conditions.

(3) Ship simulation module

The purpose of the ship simulation module is real-time presentation of the static and dynamic information about the ship in the system. This module establishes a ship model according to the static information about the ship, such as the ship type, ship width, ship length, GPS antenna position, etc., and obtains key information such as a more reliable position, sailing speed, sailing direction, ship head direction, yaw angle, etc. using a GPS, a compass, and other navigation equipment, to facilitate driving in poor visibility conditions and implement the ship dynamic modeling.

(4) Real ship driving module

The purposes of the real ship driving module are to collect nearby target ship data using an AIS and radar, input the information into a target ship simulation module after conducting classification, and then select and process the data. The ship motion data are collected using a GPS and gyrocompass and are then input into the ship simulation module. The 3D traffic environment, dynamic target ship, and dynamic ship models are integrated into the real ship driving module, which provides a multi-angle, intuitive, and selective comprehensive visual scene to assist the deck officers with operation of the ship.

### Traffic environment modeling and 3D visualization

The topographic features, channel parameters, and hydrological features of inland rivers and port water areas have distinct regional characteristics. In this study, the Hankou channel between Wuhan Yangtze River Bridge and Wuhan Yangtze River Second Bridge (between 2.5 km and 1039.1 km along the Yangtze River, corresponding to the section from its middle reaches to its lower reaches) was selected to conduct traffic environment modelling and 3D visualization. The total length of this channel is 6.8 km, which is the section with the busiest traffic in the middle reaches of the Yangtze River; the Han River flows into the Yangtze River in this channel as well. Traffic accidents such as ship collisions with bridges have occurred numerous times in this channel, its terrain and landforms have distinctive symbolic characteristics, and many ferry and cruise ships travel through it. Furthermore, the channel conditions and flow patterns are very complex, and there are many foggy days in autumn and winter, which often lead to water traffic suspension. According to the analysis of historical accident statistics [19], water traffic accidents in the Hankou channel are obviously associated with insufficient perception and analysis on the part of the deck officers of information about, for example, the underwater topography, the buildings near and crossing the river, navigation aid signs, the water flow, the wind conditions, the water depth, and dynamic information about other ships and the ship of interest itself during operation.

The endpoint coordinates of the modeling area between Wuhan Yangtze River Bridge and Wuhan Yangtze River Second Bridge are as shown in Table 1.

**Table 1:** The endpoint coordinates of the modeling area.

	Mercator coordinate (original map coordinate)	WGS84 coordinate system (latitude and longitude coordinate)
**Bottom left corner**	12721261.7434453, 3572972.1277956	114.2770386, 30.5386079
**Top left corner**	12721261.7434453, 3583368.5435815	114.2770387, 30.6190049
**Top right corner**	12728293.9500475, 3583368.5435381	114.3402100, 30.6190048
**Bottom right corner**	12728293.9500475, 3572972.1277956	114.3402100, 30.5386079

The data collected about the water area discussed in this paper are summarized in Table 2.

**Table 2:** Summary of channel space data.

Object	Parameter	Unit	Remark
**Hydrologic factors**	Topography of river bottom	m	Digital elevation
	Water depth	m	
	Depth contour	line	1 m intervals
	Flow direction		Two-dimensional (2D) vector, MAX dot matrix data processing
	Flow speed		
**Channel**	Channel boundary	9 sets	Three seasons of drought, mild weather, and flooding
	Navigational aid sign	8 pcs	5 pcs in upper reaches, 3 pcs in lower reaches
	Obstruction sign	5 pcs	Sunken ships
**Special water area/route**	Anchorage sign	2 pcs	
	Anchorage boundary line	8 lines	
	Ferry ship route	4 routes	
**Aquatic buildings**	Bridge pier	18 piers	
	Bridge pier number mark	18 pcs	8+10 (Numbers 7–16)
	Dock	52 docks	

**Fig 2:**
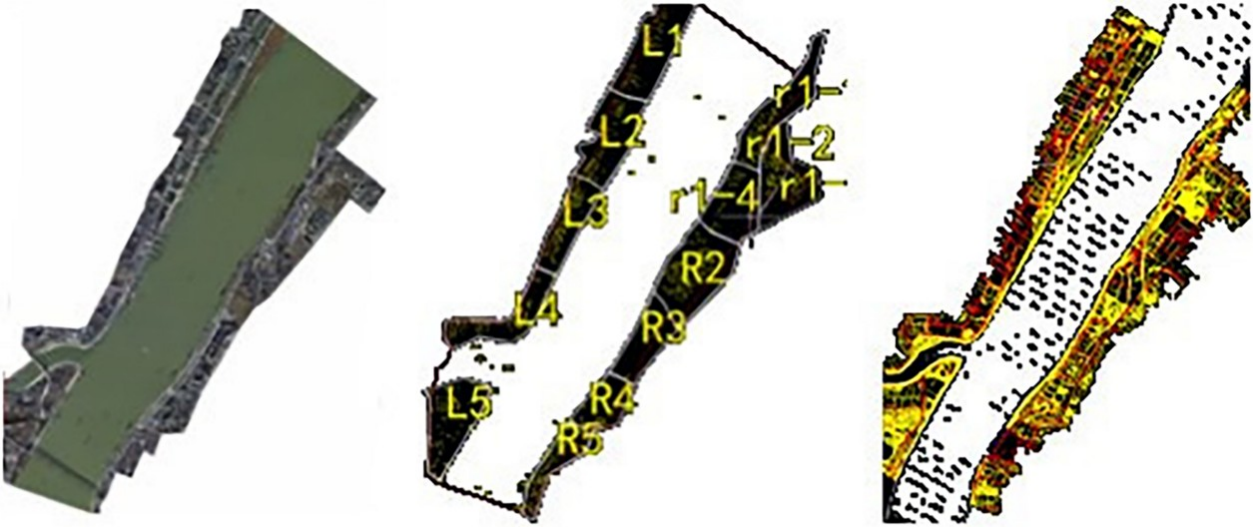
Distribution of visual parts along the river shore and orthophoto area.

The spatial information about the coastal visible section was collected by performing the following steps:

1. The orthophoto area was divided between Wuhan Yangtze River Bridge and Wuhan Yangtze River Second Bridge (as shown in Table 3 and Fig 2);
2. The photos obtained using an unmanned aerial vehicle were scanned and input into the computer by utilizing a high-precision image scanner, and each aerial photo was converted into a digital ground model based on its pixels;
3. According to the significant characteristics of the shoreline, an appropriate area was selected, the terrain was built by employing a digital elevation model grid, and the terrain in other areas was built using the simulation modeling method;
4. To eliminate the projection error caused by the tilt error of the aerial photograph and topographic fluctuations, WGS84/UTM projections, orthophotos, ground digital elevations, digital line graphics, etc. were adopted;
5. The acquired landscape photos were attached to the corresponding area by mounting, cutting, etc., and the detailed parameters and shapes of the individual objects were acquired.

Since the field studies of the coastal visible section were conducted through image acquisition, which did not involve endangered or protected species, no specific permissions were required for this case.

**Table 3:** Division of orthophoto area.

Partition number	Built-up area	Housing quantity
**L1**	0.84	68
**L2**	0.72	62
**L3**	0.52	89
**L4**	0.57	70
**L5**	0.73	54
**R1**	1.93	168
**R2**	0.92	107
**R3**	0.51	33
**R4**	0.41	60
**R5**	0.41	91
**Attached hydraulic facilities**	-	59
**Total**	7.56	861

A traffic environment can be divided into the environment above the water surface, water surface and underwater traffic environment, and target ship. The environment above the water surface includes the shore buildings, shore slope, bridges, navigation aid signs, etc. The water surface and underwater traffic environment includes the river bottom topography, 3D water body, numerical flow field, etc.

### Modeling of the environment above the water surface

**Fig 3:**
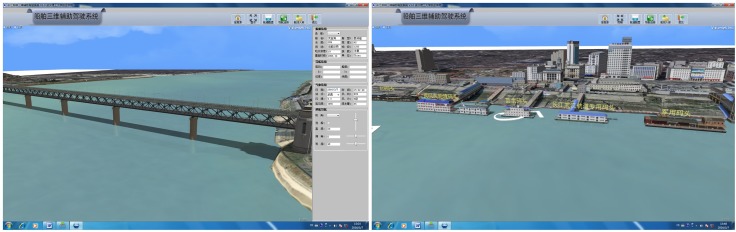
Model of the environment above the water surface.

First, 3D modeling tools such as 3Ds Max and other 3D engine platforms such as OSG or Vega are selected. Then, the channel as well as the objects and landforms along the shore are built in 3D, and the model for the static objects in the traffic environment is constructed, as shown in Fig 3.

### Modeling of the water surface and underwater traffic environment

**Fig 4:**
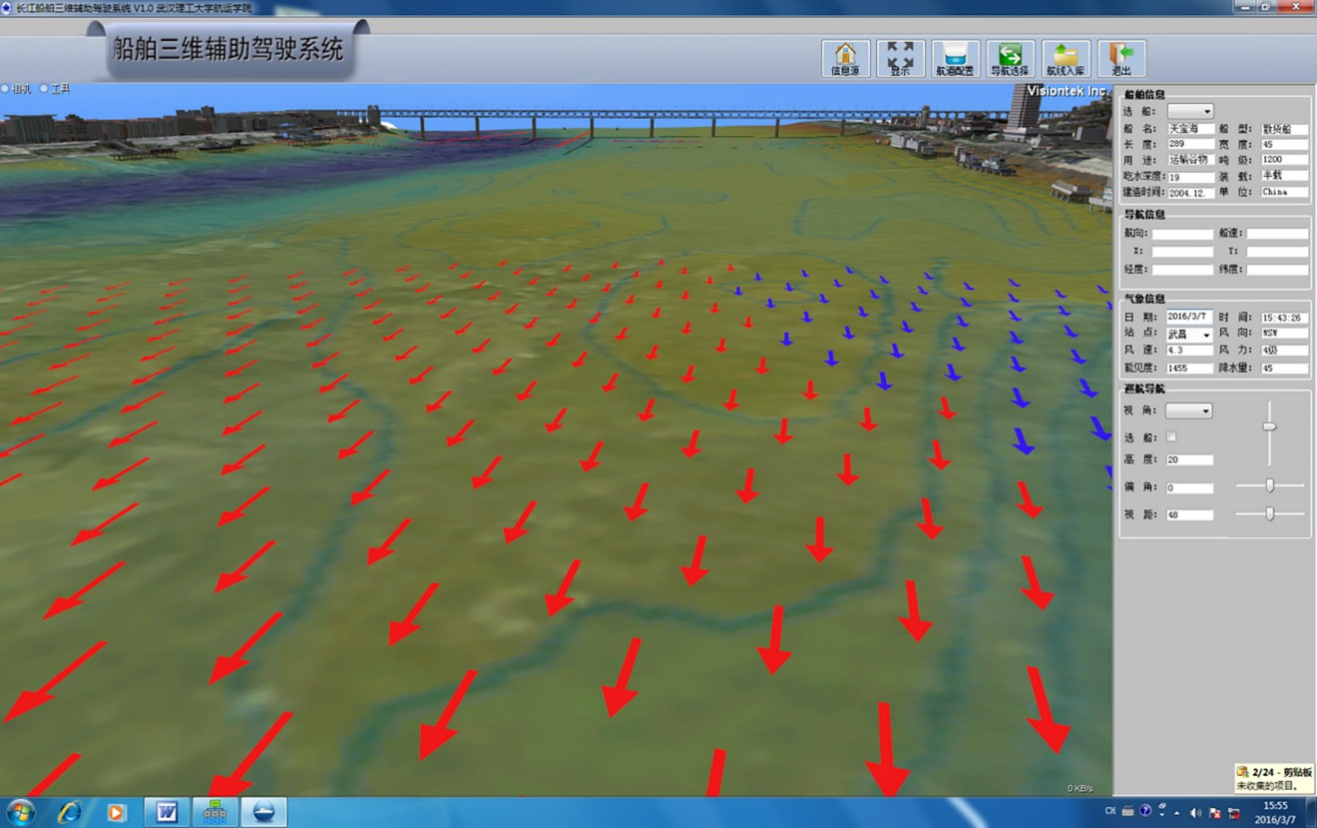
Modeling rendering of water surface and underwater traffic environment.

The channel parameters are mainly extracted and processed using image coordinate recovery, manual measurement, artificial model adjustment, and secondary modeling. Channel/navigation aid signs are marked using methods such as channel map extraction, coordinate correction, text modeling, and OSG modeling. The depth contour is generated based on data from a river bottom water depth map: the points with the same depth are connected; the water depth is manually annotated; and the original water depth data coordinate projection is transformed to establish the OSG 3D model of the underwater terrain. The generated obstructions are calibrated using artificial positions, and the model is built by OpenGL. The finite volume method and staggered grid technique are adopted in the 3D modeling. SIMPLEC and the under-relaxation technique are employed for the numerical calculations, which are verified using dynamic boundary technology by making sparse and adding the direction signs for the MAX matrix data. The flow data are displayed in the 3D visualization module as 2D vectors [14, 20, 21], as shown in Fig 4.

## Fusion of dynamic ship information

**Fig 5:**
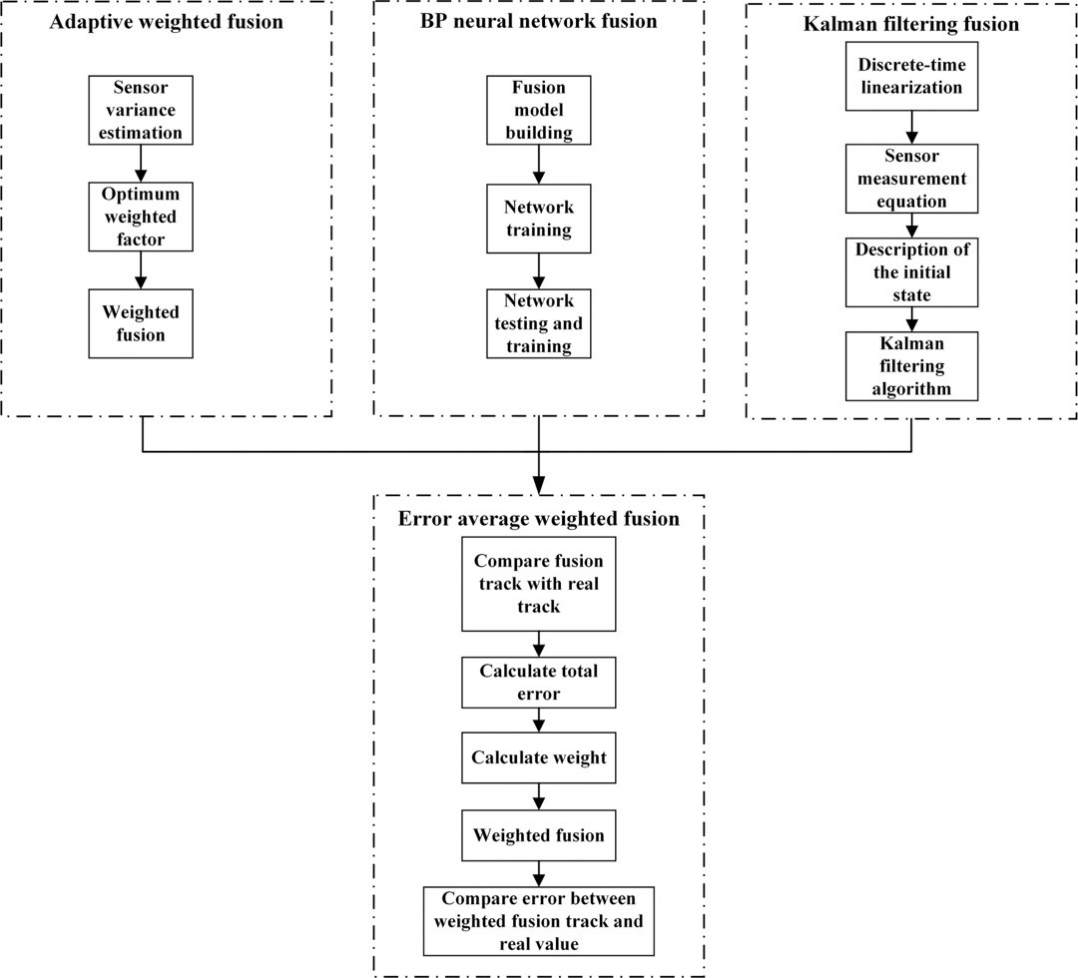
Flowchart of integrated ship dynamic information fusion technology.

When visibility is poor, the target ship information can only be acquired using two kinds of shipborne navigation equipment, radar and AIS. Furthermore, inland waterway channel conditions are complex, the channel width is limited, the ship density is high, and ships change sailing directions frequently because there are many curves. To characterize the dynamic characteristics of the target ship more accurately, integrated fusion should be conducted for radar and AIS information. Multilevel processing is the main method of information fusion and involves dividing the data fusion model into two: the functional model built according to the order of nodes and the data model built according to data extraction. In this research, a mixed model based on a control loop structure was adopted, based on the cyclic characteristics of information fusion processing, in an attempt to increase the reconstruction accuracy achieved through the processing tasks and to facilitate drawing of the location fusion behavior in the model. The methods adopted included the adaptive weighted average, BP neural network, and Kalman filter fusion algorithms, as shown in Fig 5.

### Adaptive weighted track fusion

#### Optimal weighted factor

Assuming that the true value of the target track at time *k* of the system is *X*, the values measured by the AIS and radar are *X_Ai_*(*k*) and *X_Ri_*(*k*), respectively. *X_Ai_*(*k*) and *X_Ri_*(*k*) are independent of one another and are unbiased estimates of the true value *X*. The measured error variance and weight factor of the AIS are *δ*_1_^2^ and *ω*_1_, respectively, while those of the radar are *δ*_2_^2^ and *ω*_2_, respectively, and the value after fusion is *X*. These quantities obey the following relationships (Eq 1),

$$X=\omega_{1}x_{1}+\omega_{2}x_{2}$$ (1)

and

$$1=\omega_{1}+\omega_{2}$$ (2)

The total mean square error is

$$\delta^{2}=E[{\left(x-X\right)}^{2}]\sum_{p=1}^{2}\omega_{p}^{2}{\left(x-x_{p}\right)}^{2}+2\sum_{\begin{array}{l} p=1\\ q=1\\ p\neq q \end{array}}^{2}\omega_{p}\omega_{q}(x-x_{p})(x-x_{q})$$ (3)

After deduction, when *δ*^2^ is the minimum,

$$\omega_{1}=\frac{\delta_{2}^{2}}{\delta_{1}^{2}+\delta_{2}^{2}},\;\omega_{2}=\frac{\delta_{1}^{2}}{\delta_{1}^{2}+\delta_{2}^{2}}$$ (4)

At this time, $\delta^{2}=\frac{\delta_{1}^{2}\delta_{2}^{2}}{\delta_{1}^{2}+\delta_{2}^{2}}$

#### Weighted fusion

If the measurement variance and weighted factors of AIS and radar are known, the variance, distance, azimuth, ground speed, and sailing direction after fusion can respectively be obtained using

$$\delta_{X_{ij}(k)}^{2}=\frac{\delta_{ad}^{2}\delta_{rd}^{2}}{\delta_{ad}^{2}+\delta_{rd}^{2}}$$ (5)

$$\delta_{Y_{ij}(k)}^{2}=\frac{\delta_{a\theta }^{2}\delta_{r\theta }^{2}}{\delta_{a\theta }^{2}+\delta_{r\theta }^{2}}$$ (6)

$$X_{ij}(k)=\omega_{ad}X_{Ai}(k)+\omega_{rd}X_{Ri}(k)=\frac{\delta_{rd}^{2}}{\delta_{ad}^{2}+\delta_{rd}^{2}}X_{Ai}(k)+\frac{\delta_{ad}^{2}}{\delta_{ad}^{2}+\delta_{rd}^{2}}X_{Ri}(k)$$ (7)

and

$$Y_{ij}(k)=\omega_{a\theta }Y_{Ai}(k)+\omega_{r\theta }Y_{Ri}(k)=\frac{\delta_{r\theta }^{2}}{\delta_{a\theta }^{2}+\delta_{r\theta }^{2}}Y_{Ai}(k)+\frac{\delta_{a\theta }^{2}}{\delta_{a\theta }^{2}+\delta_{r\theta }^{2}}Y_{Ri}(k)$$ (8)

### BP neural network fusion

#### Establishment of fusion model

The network input and output, errors of the hidden nodes and network target, training times, and learning rate are set up and trained by adding training data. After constructing the fusion model, the network is trained so that it meets the setup requirements. Then, the associated AIS and radar data are input to obtain the merged information that forms the network output.

#### Network testing and verification

After training the network by extracting the AIS data as well as the first *n* sets of radar data, the trained network is tested and verified. This paper discusses network evaluation using the formula $\bar{E_{k}}=\frac{1}{N}\sum_{i=1}^{N}\left|D_{k}^{F}(i)-D_{k}^{I}(i)\right|$.

In the formula, found represennts the *k*th characteristic value after fusion at time *i*, *F* represents the data after BP fusion, found represennts the *k*th real characteristic value of the real ship at time *i*, and *I* represents the real ship data.

To evaluate the fusion results, the formulae $\bar{B_{k}}=\frac{1}{N}\sum_{i=1}^{N}\left|D_{k}^{A}(i)-D_{k}^{I}(i)\right|$ and $\bar{C_{k}}=\frac{1}{N}\sum_{i=1}^{N}\left|D_{k}^{R}(i)-D_{k}^{I}(i)\right|$ are used to represent the differences between the AIS and real values and between the radar and real values, respectively.

In the formula, *D_k_^A^*(*i*) found represennts the *k*th characteristic AIS value at time *i*, *A* represents the AIS data, found represennts the *k*th real characteristic radar value at time *i*, and *R* represents the radar data.

### Kalman filtering fusion

The basic principle of radar and AIS fusion involves combining the information from the two sensors in space and time according to a criterion to generate more reasonable and accurate data than could be obtained by a single sensor. The Kalman state estimate can be employed to smooth the radar and AIS data, filter the measured ship data, and predict the future track of the ship. Kalman filter theory was adopted using the time domain method to obtain the optimal estimate according to the state equation.

The state estimation of the target during data fusion can be expressed as

$$\begin{array}{l} \overset{⌢}{X}(k+1|k+1)=[I-K(k+1)H(k+1|k)]X(k+1|k)+\\ K(k+1)Z(k+1)=I-K(k+1)H(k+1)=P(k+1|k+1)P{\left(k+1|k\right)}^{-1} \end{array}$$ (9)

For both radar and AIS,

$$\begin{array}{l} 1-I-P_{i}{\left(k+1|k+1\right)}^{-1}H_{i}(k+1{)}^{\prime }R_{i}{\left(k+1\right)}^{-1}H_{i}(k+1)\\ =P_{i}(k+1|k+1)P_{i}{\left(k+1|k\right)}^{-1} \end{array}$$ (10)

and

$$\begin{array}{l} H_{i}(k+1{)}^{\prime }R_{i}{\left(k+1\right)}^{-1}Z_{i}(k+1)=\\ P_{i}{\left(k+1|k+1\right)}^{-1}{\overset{⌢}{X}}_{1}(k+1|k+1)-P_{i}{\left(k+1|k+1\right)}^{-1}{\overset{⌢}{X}}_{1}(k+1|k) \end{array}$$ (11)

It can be determined that

$$\begin{array}{l} {\overset{⌢}{X}}_{1}(k+1|k+1)=P(k+1|k+1)\{P_{i}{\left(k+1|k\right)}^{-1}{\overset{⌢}{X}}_{1}(k+1|k)+\\ {\sum_{i=1}^{2}P_{i}(k+1|k+1)}^{-1}{\overset{⌢}{X}}_{1}(k+1|k+\text{1})-P_{i}{\left(k+1|k\right)}^{-1}{\overset{⌢}{X}}_{1}(k+1|k)\} \end{array}$$ (12)

$$\overset{⌢}{X}(k+1|k+1)=\Phi (k)\overset{⌢}{X}(k|k)$$ (13)

$$\begin{array}{l} P_{i}{\left(k+1|k+1\right)}^{-1}=P_{i}{\left(k+1|k\right)}^{-1}+\\ \sum_{i=1}^{2}P_{i}{\left(k+1|k+1\right)}^{-1}-P_{i}{\left(k+1|k\right)}^{-1} \end{array}$$ (14)

and

$$P(k+1|k)=\Phi (k)P(k|k)\Phi (k{)}^{\prime }+G(k)Q(k)G(k{)}^{\prime }$$ (15)

Where *X*(*k*|*k*) represennts the state vector of the target at time k, $\overset{⌢}{X}(k|k)$ represennts the estimation of the target state vector at time k, *K*(*k*) represennts the filter gain at moment k, *H*(*k*) represents the observation matrix at the time k, *P*(*k*|*k*) represents the covariance at time k, *Z*_*i*_(*k*) represents the measurement residuals, Φ(*k*) represents state transfer matrix, *G*(*k*) represents the noise distribution matrix, *Q*(*k*) represents positive definite covariance matrix.

### Integrated fusion

To improve the stability and reliability of the system, data fusion adopt multi-source heterogeneous data fusion model which integrates the methods of Calman filtering, adaptive weighting and neural network, etc. The error of fusion data and real value that the different methods draw may differ greatly. The errors of data fusion and real data of three methods are respectively compared to obtain the weight of three fusion method by the weighted average method, and the final fusion data is obtained. The weight of the three fusion methods can be obtained by the weighted average formula.

According to the weighted average formula:

The *x*- and *y*-coordinate weights of the adaptive weighted, BP neural network, and Calman filtering fusion methods are $\omega_{x1}=\frac{\sigma_{2}+\sigma_{3}}{2(\sigma_{1}+\sigma_{2}+\sigma_{3})}$ and $\omega_{y1}=\frac{\delta_{2}+\delta_{3}}{2(\delta_{1}+\delta_{2}+\delta_{3})}$, $\omega_{x2}=\frac{\sigma_{1}+\sigma_{3}}{2(\sigma_{1}+\sigma_{2}+\sigma_{3})}$ and $\omega_{y2}=\frac{\delta_{1}+\delta_{3}}{2(\delta_{1}+\delta_{2}+\delta_{3})}$, and $\omega_{x3}=\frac{\sigma_{1}+\sigma_{2}}{2(\sigma_{1}+\sigma_{2}+\sigma_{3})}$ and $\omega_{y3}=\frac{\delta_{1}+\delta_{2}}{2(\delta_{1}+\delta_{2}+\delta_{3})}$ respectively.

Then, track fusion can be represented as

$$x_{r}(k)=\frac{\sigma_{2}+\sigma_{3}}{2(\sigma_{1}+\sigma_{2}+\sigma_{3})}.x_{aw}(k)+\frac{\sigma_{1}+\sigma_{3}}{2(\sigma_{1}+\sigma_{2}+\sigma_{3})}.x_{BP}(k)+\frac{\sigma_{1}+\sigma_{2}}{2(\sigma_{1}+\sigma_{2}+\sigma_{3})}.x_{km}(k)$$ (16)

and

$$y_{r}(k)=\frac{\delta_{2}+\delta_{3}}{2(\delta_{1}+\delta_{2}+\delta_{3})}.y_{aw}(k)+\frac{\delta_{1}+\delta_{3}}{2(\delta_{1}+\delta_{2}+\delta_{3})}.y_{BP}(k)+\frac{\delta_{1}+\delta_{2}}{2(\delta_{1}+\delta_{2}+\delta_{3})}.y_{km}(k)$$ (17)

where *x_aw_*(*k*), *x_BP_*(*k*), and *x_km_*(*k*) represent the *x*-coordinates of the adaptive weighted, BP neural network, and Calman filtering fusion track points, respectively, at time *k*, and *y_aw_*(*k*), *y_BP_*(*k*), and *y_km_*(*k*) represent the y-coordinates of adaptive weighted, BP neural network, and Kalman filtering fusion track points, respectively at time *k*.

## System construction and real ship verification

### System construction

**Fig 6:**
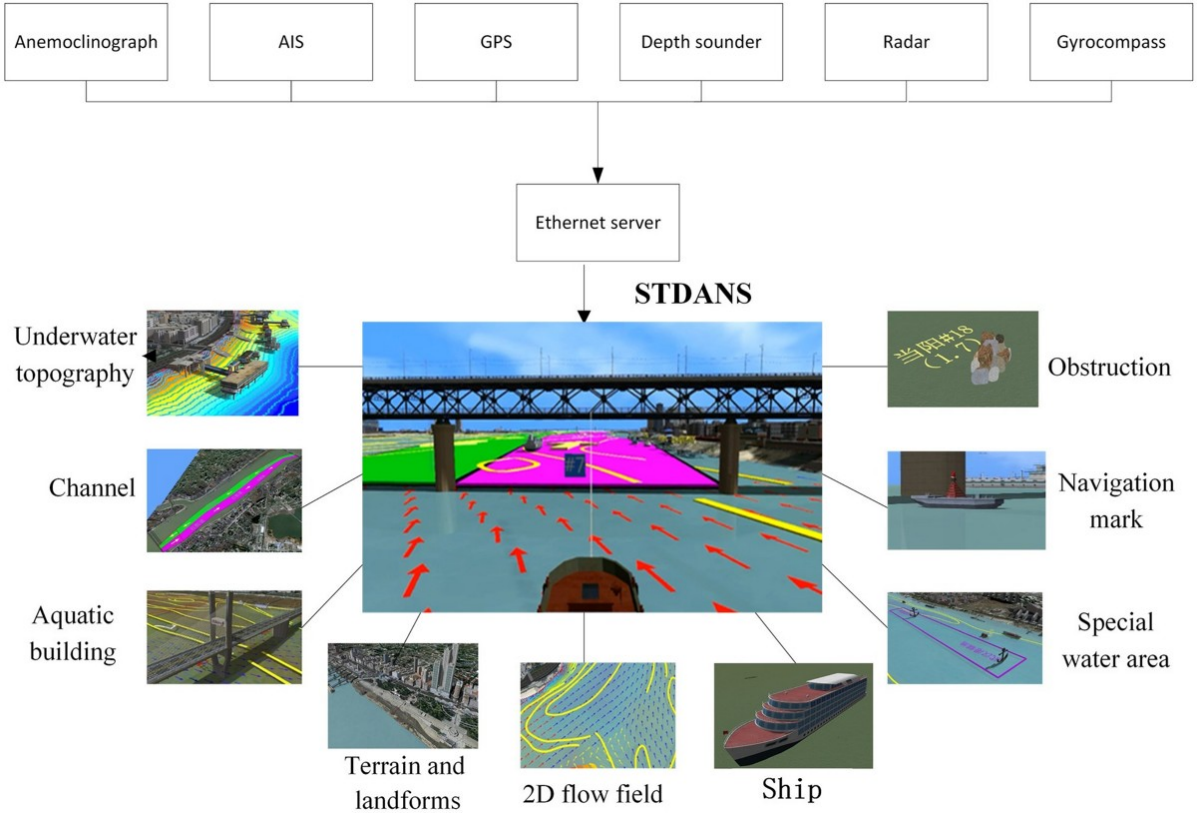
Schematic diagram of STDANS construction.

The ship and target ship in the STDANS are driven using real navigational aid data, as shown in Fig 6. The upper half of the figure illustrates the shipborne equipment used to input the static and dynamic parameters of the ship and target ship into the STDANS. The lower half depicts the construction of a 3D traffic scene.

### Real ship trial

The real ship trial was conducted on April 17, 2014 using a patrol ship for the STDANS principle prototype test. The verification was performed by driving the ship in water and collecting real traffic environment data, then obtaining simulated traffic environment data using the STDANS and comparing and analyzing the results to verify the accuracy and reliability of the STDANS. The experimental equipment loaded onto the ship, which was called Sea Patrol 12319, included two STDANS hosts, an HCM365 all-attitude 3D electronic compass, a GPS terminal, a DGPS terminal, and a shipborne AIS terminal. As the target ship, the ferry called Jiangcheng No. 11 had one DGPS terminal. Its antenna was located near the GPS antenna that was already on the ferry, and the DGPS data were utilized as reference ship position data.

**Fig 7:**
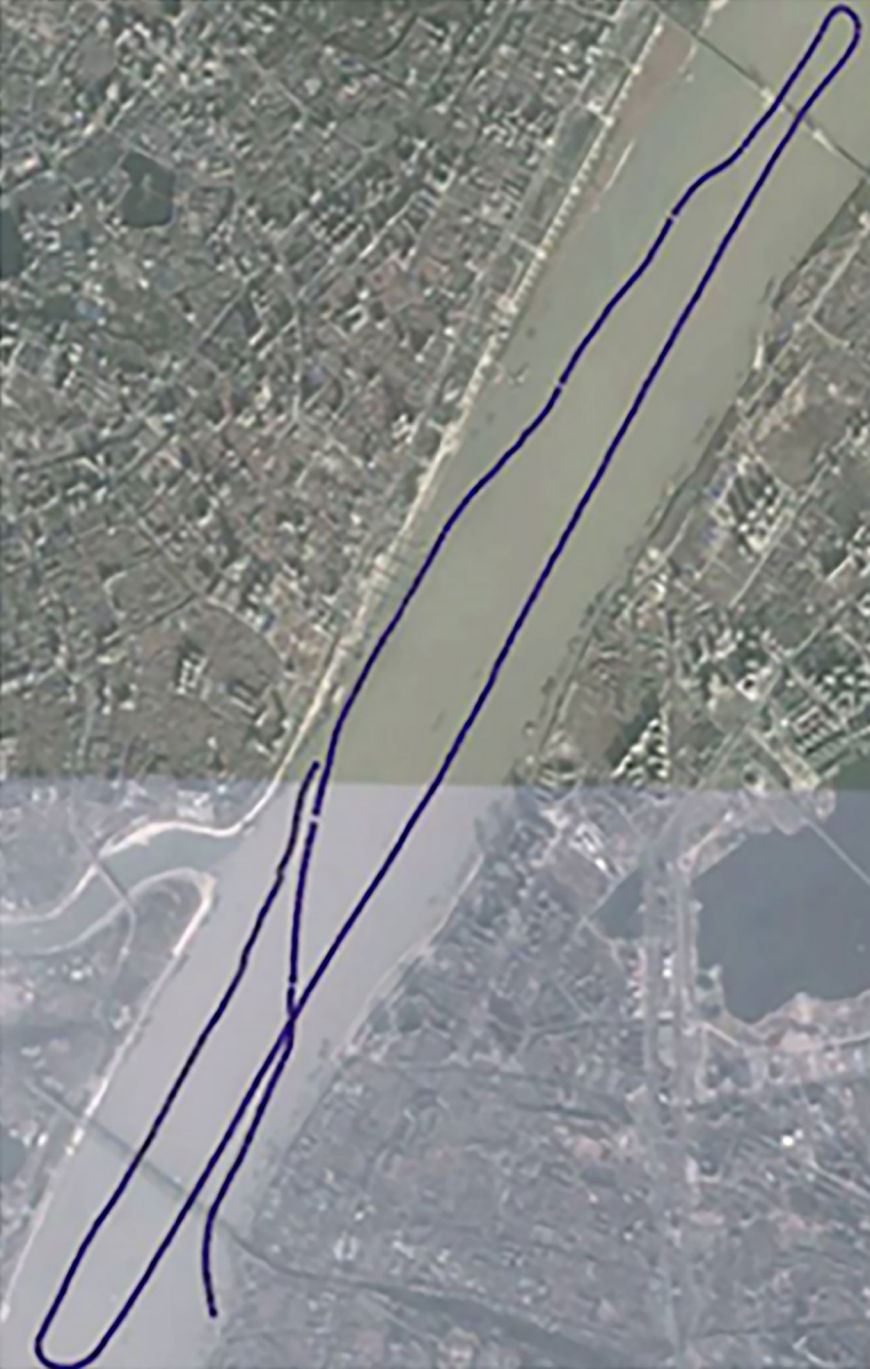
Trial track of the real ship.

The patrol ship set out from the maritime port of Wuhan customs on the north shore of the Yangtze River, sailed upstream, passed through the Wuhan Yangtze River Bridge, made a U-turn, and sailed downstream. It then passed under the Wuhan Yangtze River Bridge and Wuhan Yangtze River Second Bridge, made a U-turn again, sailed upstream, and stopped at the Pinghumeng marine port on the south shore of the Yangtze River. The whole course included two U-turns and multiple speed and direction variations. The trial track of the sea patrol ship and real ship is shown in Fig 7. In the STDANS, real ship verification includes static and dynamic tests, which can be divided into three stages: preparation, operation, and data validation. The details of the experimental plan in each stage are shown in Table 4.

**Table 4:** Experimental plan for a real ship in the STDANS.

Stage		Work content		Expected time	Achievement confirmation
**Preparation**	1.1	Work handover	1. Submit a list for this activity; 2. Specify the sailing route and points for attention (sailing direction, speed, points of identification); 3. The route distance is about 50 min, and the route length is 10–20 km.	5 min	
	1.2	Environment configuration	1. Ship power supply: 220V AC; 2. Deployment of the station: AIS, GPS installation deployment location, STDANS operation, power and fixed equipment, etc.	20 min	If completed
	1.3	Connectivity test	1. Start up main server verification, and deploy standby server verification; 2. GPS data preparation (GPS must be external, warm boot is reserved for 10 min) and AIS signal verification (synchronous); 3. Identify the trial ship and perform ID locking; 4. Confirm the route and departure time.	15 min	Complete starting
**Operation**	2.1	Sailing record	1. Record the sailing data along the whole course, and verify the location of the test data online.	50 min	Online monitor
	2.2	Confirm the trial ship	2. Confirm the sailing position, and identify and verify relevant ship information.		
	2.3	Object and ground verification	3. Make comparison to the core markers, and confirm the relationship to the real scene (take photos and compare); 4. Ensure that the track is correct and the task is terminated.		
**Data validation**	3.1	Track reconstruction	1. Export data and display track.		Analyze and report
	3.2	Route confirmation	2. Compare the photos.		
	3.3	Simulation play-back	3. Analyze and report.		

### Validation

#### Trial ship

Because of the high positioning accuracy of DGPS, the tracks output by two groups of terminals were analyzed and compared to the DGPS track, and it was determined that the STDANS prototype could accurately reflect the sailing track of the trial ship.

The trial ship motion was analyzed based on the fore- and aft-direction trial ship data that were acquired in real time and displayed on the screen of the STDANS in typical conditions, including straight line navigation, steering, mooring, etc. Based on the fore- and aft-direction data measured by the electronic compass, the error variance was calculated to evaluate the perception ability provided to deck officers in complex weather conditions by the STDANS, and the results are shown in Table 5.

**Table 5:** Error calculation data.

Number	Sailing pattern	Navigation equipment	Average error	Range error	Error variance
**1**	Straight upstream	Magnetic compass	3.45°	6.6	1.97
		GPS	4.75°	7.2	1.92
**2**	Straight downstream	Magnetic compass	2.25°	6.9	1.22
		GPS	4.01°	9.4	2.18
**3**	U-turn in upper reaches	Magnetic compass	2.25°	4.8	1.00
		GPS	4.01°	9.3	6.30
**4**	U-turn in lower reaches	Magnetic compass	4.48°	6.4	3.46
		GPS	8.59°	10	9.97
**5**	Sailing with slight direction variation	Magnetic compass	1.14°	3	0.24
		GPS	4.62°	3.6	0.78
**6**	Mooring	Magnetic compass	4.98°	3.9	1.14
		GPS	32.19°	190	2513.05

By letting *δ_Mi_* and *δ_Gi_* represent the magnetic compass and GPS measurement errors, respectively, the magnetic compass and GPS stabilities can be respectively expressed as:

$$S_{M}=\sqrt{\frac{{\left(\delta_{M1}-\bar{\delta_{M}}\right)}^{2}+{\left(\delta_{M2}-\bar{\delta_{M}}\right)}^{2}+\ldots +{\left(\delta_{Mn}-\bar{\delta_{M}}\right)}^{2}}{n}}$$ (18)

$$\mathrm{and}\;S_{G}=\sqrt{\frac{{\left(\delta_{G1}-\bar{\delta_{G}}\right)}^{2}+{\left(\delta_{G2}-\bar{\delta_{G}}\right)}^{2}+\ldots +{\left(\delta_{Gn}-\bar{\delta_{G}}\right)}^{2}}{n}}$$ (19)

The error stabilities in different sailing conditions are summarized in Table 6.

**Table 6:** Error stability.

Sailing condition number	1	2	3	4	5	6
**Stability (magnetic compass)**	1.40	1.10	1.00	1.86	1.36	0.92
**Stability (GPS)**	1.69	1.48	3.77	3.08	1.73	61.30

From the above calculations and analysis, it can be seen that there are certain degrees of average error, error range, and error variance in different sailing conditions for the fore- and aft-direction data of the trial ship (GPS sailing direction) displayed on the STDANS screen. When sailing in a straight line or with a slightly varying direction, the average error, error range, and error variance of the GPS sailing direction are small, and the stability is good. In such situations, the trial ship visual scene displayed on the STDANS reflects the motion state of trial ship, which can generally meet the perception requirements of deck officers in complex weather conditions.

When performing a U-turn or undergoing significant direction changes, the average error, error range, and error variance of the GPS sailing direction are large, and the stability is poor. In these situations, the trial ship visual scene displayed on the STDANS can only roughly reflect the motion state of the trial ship, which can fundamentally meet the perception requirements of deck officers in complex weather conditions when the ship density is low.

In the situations in which the trial ship was moored or anchored, the average error, error range, and error variance of the GPS sailing direction are significantly higher and the stability is deteriorated. In these cases, the trial ship visual scene displayed on the STDANS has a deviation of almost 180° with respect to the actual state of the ship and therefore cannot reflect the motion state of the trial ship.

#### Static traffic environment comparison

**Fig 8:**
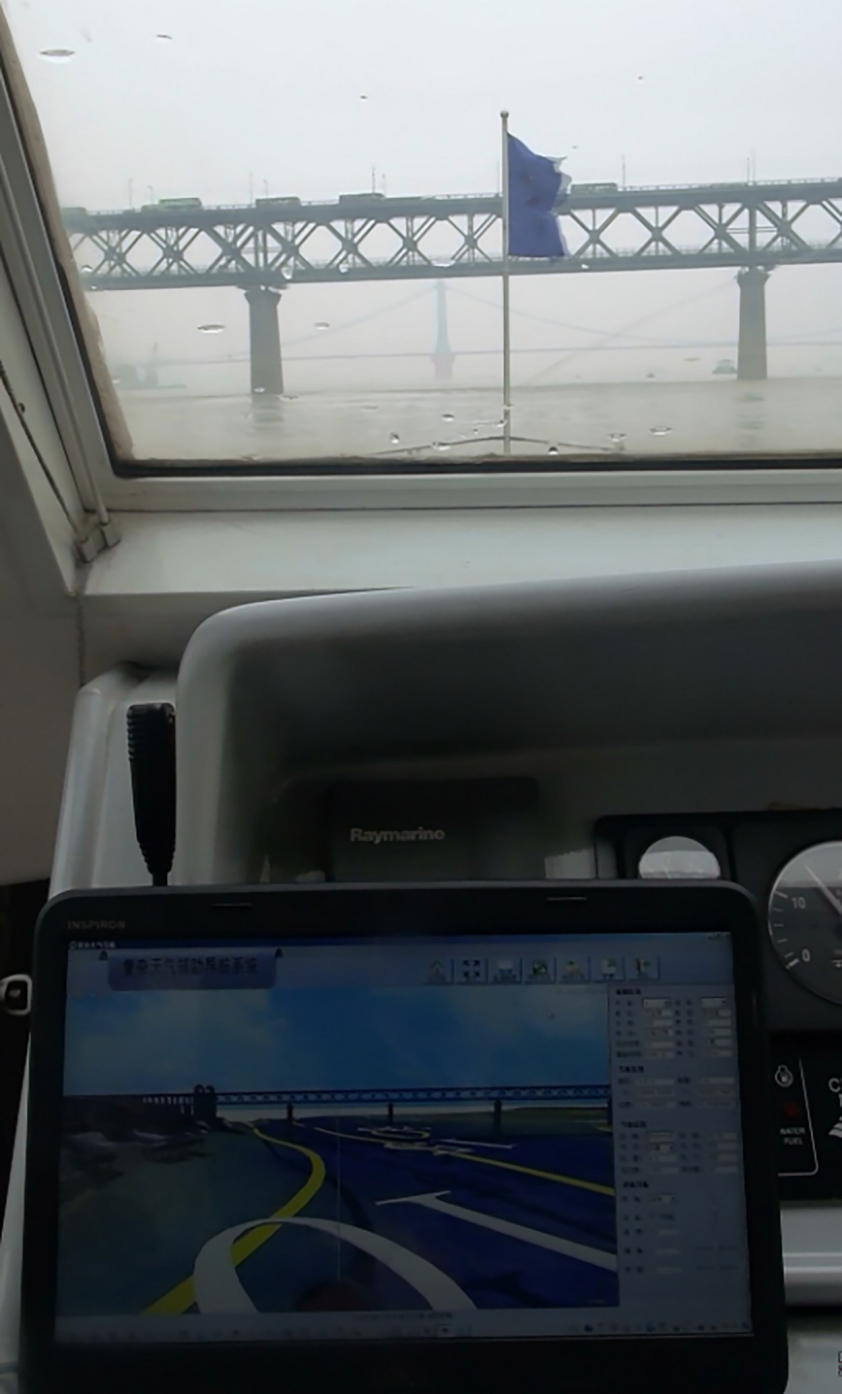
Visual scene comparison of fixed structure.

Comparison and analysis of the static traffic environment is mainly done by selecting fixed landmarks in the experimental river section (bridges, ground buildings, fixed piers, etc.). The real-time visual scene of the fixed building in the STDANS was compared with the visual scene shot by the ship called Sea Patrol 12319 in the same direction, and the accuracy was analyzed. The associated visual scenes are depicted in Fig 8 for comparison.

The comparison with the real navigation operation and analysis revealed that the STDANS prototype could display the static traffic environment more rapidly and accurately. The problems are mainly related to two aspects: first, due to the monitor, some differences exist between the viewing angles in the visual scene displayed on the STDANS screen and what human eyes observe; secondly, there are slight deviations between the angles of fixed buildings and the trial ship in the STDANS display and what the human eyes observe. The main reasons for these differences are system delay and the difference between the GPS sailing direction and the fore and aft directions of the trial ship. However, the system can still provide reliable fixed references for deck officers sailing in complex weather conditions.

#### Analysis of error fluctuation and stability of track fusion error

The target ship motion state was analyzed based on real-time DGPS target ship position acquisition. The target ship position information was simultaneously collected using shipborne radar and an AIS and input into the STDANS; the integrated data fusion model was adopted to calculate the position of the target ship; the target ship was displayed on the STDANS screen; and the integrated fusion error volatility and track fusion error stability were calculated based on the DGPS position, to test the ability of deck officers to perceive the target ship in complex weather conditions using the STDANS.

**Fig 9:**
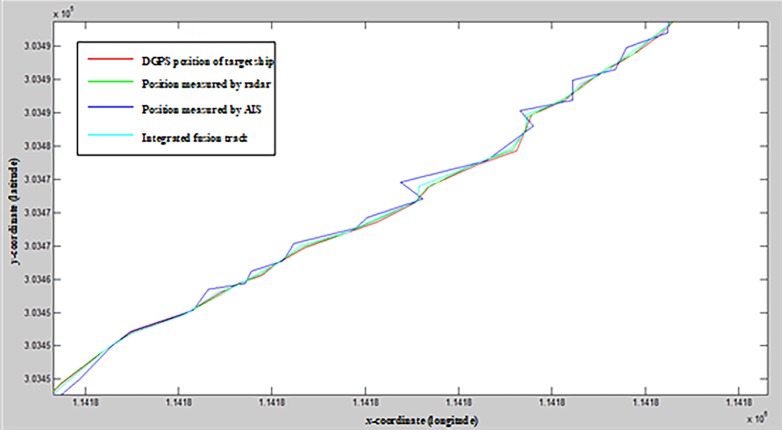
Integrated data fusion track.

According to the integrated data fusion model, the final fusion track presented in Fig 9 was calculated.

The error volatility of this model is the degree of consistency between the points on the integrated fusion track and those on the real track. The volatility of the error between these points was analyzed to obtain the error volatility of the model, which is represented by the standard deviation of the error between the points on the two tracks. The track fusion stability represents the calculated amplitude of the variation between two adjacent points in this model in the same conditions, which is represented by the standard deviation of the difference between the two points.

The coordinates of the adaptive weighted, BP neural network, Calman filtering, integrated fusion, and real track points at time *k* are defined as (*x*_*aw*_(*k*), *y*_*aw*_(*k*)), (*x*_*BP*_(*k*), *y*_*BP*_(*k*)), (*x*_*km*_(*k*), *y*_*km*_(*k*)), (*x*_*r*_(*k*), *y*_*r*_(*k*)), and (*x*(*k*), *y*(*k*)) respectively, and the error volatility is the fusion accuracy. When the error volatility is low, the fusion accuracy is high. The formula for error volatility is

$$B=\sqrt{\frac{{\left(\delta_{k}-\bar{\delta }\right)}^{2}}{n-1}}$$ (20)

where $\delta_{k}=\sqrt{{\left(x_{r}(k)-x(k)\right)}^{2}+{\left(y_{r}(k)-y(k)\right)}^{2}}$.

*δ_k_* represents the error between points on the integrated fusion track and those on the real track at the time *k*, $\bar{\delta }$ *δ_k_*, (*x_r_*(*k*), *y_r_*(*k*)) is the fusion track point at time *k*, and (*x*(*k*), *y*(*k*)) is the real track point at time *k*. *B*is the accuracy of the fusion, of which the values are shown in Table 7.

The error stability of track fusion is the sum of the difference Δ*_k_* between adjacent track points using the equations (Eqs 21 and 22) below.

$$\Delta_{k}=\sqrt{{\left(x_{k}-x_{k-1}\right)}^{2}-{\left(y_{k}-y_{k-1}\right)}^{2}}$$ (21)

$$\sum_{k=2}^{n}\Delta_{k}=\sum_{k=2}^{n}\sqrt{{\left(x_{k}-x_{k-1}\right)}^{2}-{\left(y_{k}-y_{k-1}\right)}^{2}}$$ (22)

**Table 7:** Values of *B*.

*B*_*aw*_	*B*_BP_	*B*_*km*_	*B*_j_
0°0’0.22”	0°0’0.34”	0°0’0.53”	0°0’0.18”

Where Baw *B*_*aw*_ is the accuracy of the adaptive weighted fusion; *B*_BP_ is the accuracy of the BP neural network fusion; *B*_*km*_ is the accuracy of the Kalman Filtering fusion; *B*_j_ is the accuracy of the integrated fusion.

The results of the integrated fusion error volatility calculations are as follows: *B*_*aw*_ = 133.9834, *B*_BP_ = 762.5491, *B*_*km*_ = 314.2769, and *B*_j_ *=* 4.3978. It can be seen that the integrated fusion error volatility is greatly improved.

The results of the integrated track fusion error stability calculations are as follows: $\sum_{k=2}^{n}\Delta_{kaw}$ = 865.6005, $\sum_{k=2}^{n}\Delta_{kBP}$ = 877.1249, $\sum_{k=2}^{n}\Delta_{kkm}$ = 879.4183, and $\sum_{k=2}^{n}\Delta_{kj}$ = 869.9175. It can be seen that the error stability of the integrated fusion method is better than that of the single fusion method.

Based on the calculations and analysis presented above, it can be concluded that the STDANS can improve the ability of deck officers to analyze the motion of a target ship when visibility is poor after processing the target ship data.

## Evaluation of STDANS

In order to evaluating the reliability of STDANS, a quantitative method is used through calculating the improvement rate(IR) for integrated fusion error volatility *B*_j_, the optimization rate (OR) for the error stability of the integrated fusion $\sum_{k=2}^{n}\Delta_{kj}$, as well as the bearing error (BR) between real fixed target and its simulated target. IR and OR on adaptive weighted track fusion can be respectively achieved in Eq 23 and Eq 24.

$$IR_{aw}=\frac{1}{N}\sum_{i=1}^{N}\frac{B_{j}-B_{aw}}{B_{aw}}\times 100\%$$ (23)

$$OR_{aw}=\frac{1}{N}\sum_{i=1}^{N}\frac{{△}_{kj}-{△}_{kaw}}{{△}_{kaw}}\times 100\%$$ (24)

BR can be calculated using Eq 25.

$$BR_{i}=\sqrt{\frac{{\left(C_{si1}-C_{fi1}\right)}^{2}+{\left(C_{si2}-C_{fi2}\right)}^{2}+K+{\left(C_{sin}-C_{fin}\right)}^{2}}{n}},\;i=1,2,\cdots ,n$$ (25)

The real ship trial is carried out in six sailing conditions, including straight upstream, straight downstream, U-turn in upper reaches, U-turn in lower reaches, sailing with slight direction variation as well as mooring. Outcome of IR and OR for the six sailing conditions is shown as Table 8.

**Table 8:** Outcome of IR and OR for six sailing conditions.

Sailing condition number			1	2	3	4	5	6
***B*_j_**			0°0’0.21”	0°0’0.15”	0°0’0.79”	0°0’1.11”	0°0’0.38”	0°0’6.13”
**Improvement rate (IR) for error volatility (%)**		*aw*	52.0	31.7	23.9	47.0	35.3	14.8
		*BP*	37.4	67.6	39.6	44.2	18.3	-33.7
		*km*	49.1	46.4	19.5	32.9	10.8	6.0
$\sum_{k=2}^{n}\Delta_{kj}$			634.9637	801.4171	967.0018	1009.9170	849.4575	2044.8136
**Optimization rate(OR) for error stability** **(%)**		*aw*	6.4	-3.7	2.9	-1.4	3.3	1.1
		*BP*	3.9	6.6	3.6	4.2	-1.8	-3.1
		*km*	-2.8	4.4	-1.5	3.9	1.7	0.5

Meanwhile, in the real ship trial, three fixed targets are selected for measuring their bearings in real time, including the upstream white No.1 buoy for Bridge, the downstream red No.2 buoy for Bridge, as well as Guishan TV Tower.

Outcome of BR between the three real fixed targets and their simulated points in STDANS is shown as Table 9.

**Table 9:** Outcome of *BR* between real and virtual targets.

Measuring points		1	2	3	4	5	6	7	8	9	10	11	12
***BR* for upstream white No.1 buoy for Bridge**	*r*	10.7	18.4	24.2	39.9	57.9	65.5	71.3	96.1	102.0	135.8	148.6	162.4
	*v*	10.7	18.4	24.2	39.9	57.8	65.5	71.3	96.1	102.0	135.8	148.5	162.4
	*e*	0	0	0	0	-0.1	0	0	0	0	0	-0.1	0
	*BR*	0.0408											
***BR* for downstream red No.2 buoy for Bridge**	*r*	11.9	18.0	26.1	42.8	62.4	71.7	85.2	98.5	107.6	124.8	145.0	167.2
	*v*	11.9	18.1	26.1	42.8	62.4	71.6	85.2	98.5	107.6	124.9	145.0	167.2
	*e*	0	0.1	0	0	0	-0.1	0	0	0	0.1	0	0
	*BR*	0.02887											
***BR* for Guishan TV Tower**	*r*	0	15	30	45	60	75	90	105	120	135	150	165
	*v*	0	15	30	45	60	75	90	104.9	120	135	150	165
	*e*	0	0	0	0	0	0	0	-0.1	0	0	0	0
	*BR*	0.05											

Based on the above reliability evaluation of STDANS, when ship is sailing, the system can accurately describe the static objects beforehand and recreate the dynamic objects with good outcome in real time.

## Conclusion

The 3D visualization platform integrates hydrological and meteorological, terrain and landform, channel, ship, and sensor information, meanwhile visually demonstrates the traffic environment above the water through AIS data. Visualization of the traffic environment provides assistance for the ship driver to make decisions, especially under complex weather conditions such as poor visibility. This paper has proposed the idea of systematically integrating simulations and real ship sailing, clarified the characteristics that a STDANS should possess, described the overall system framework and module design, and presented research on ship navigation theory and the method of “virtual–reality” and “dynamic–static” combination, including experimental verification. The work presented in this paper can be summarized as follows:

1. Poor visibility prevents ship crews from obtaining necessary traffic information, hindering and restricting the abilities of deck officers to perceive and analyze their traffic environments and ship states, which directly affect the ship navigation safety and traffic efficiency. Complex weather is frequent, variable, paroxysmal, and risky. Therefore, as a highly integrated system, the STDANS should involve simulation, fusion, integration, and reliability and should be composed of four modules: traffic environment simulation, target ship simulation, ship simulation, and real ship driving. The key points are solving the main issues of traffic environment modelling, 3D visualization, information fusion, and real ship driving integration.
2. In this study, the channel section between Wuhan Yangtze River Bridge and Wuhan Yangtze River Second Bridge was selected as an example and environmental modelling and 3D visualization research was conducted. High-precision channel, land terrain, and aquatic building data were combined with 3D modelling tools such as 3D MAX and 3D engine platforms such as OSG or Vega were selected to build the 3D waterway and coastal topography, illustrating the real features of the water area and terrain between Wuhan Yangtze River Bridge and Wuhan Yangtze River Second Bridge. Furthermore, related 3D channel object elevation information was integrated. The hydrological and meteorological numerical modelling was performed using the finite volume method and staggered grid technique to discretize the control equation, and the numerical calculations were conducted using SIMPLEC and under-relaxation technique. The flow field data was displayed in a 3D visualization module in the form of 2D vectors by converting the MAX dot matrix data.
3. This paper presented a multi-source heterogeneous data fusion model for integrating AIS and radar data, in which the Kalman filtering, adaptive weighted, and neural network methods are adopted to conduct integrated fusion. This technique also yields improved error volatility, thereby enhancing the track fusion error stability to a certain extent.
4. Research on system realization and real ship verification was performed. The ship navigation equipment signal was input into the STDANS to control the sailing of a trial ship in the channel section between the Wuhan Yangtze River Bridge and Wuhan Yangtze River Second Bridge. The system operation effects and real traffic environment were compared. The results of the real ship trial verified the principle and feasibility of the proposed system.

Subsequent research on STDANSs will focus on:

1. Conducting real-time numerical simulations of 3D flow fields that are mixed with real measured flow field data, to improve the precision, accuracy, and speed of the environmental field simulation further.
2. Operating shipborne AISs in group assigning mode by controlling the base stations sending AIS messages and improving the AIS data refresh rate. The DGPS monitoring station will be set up at the same location as the AIS base station, and the ship positioning accuracy will be corrected and improved using the base station broadcasts.
3. Integrating the environment field information into the dynamic ship information fusion model and further strengthening the system integration of the model.
